# Cholera surveillance time series in Africa between 2010 and 2023

**DOI:** 10.12688/gatesopenres.16378.1

**Published:** 2026-04-14

**Authors:** Qulu Zheng, Rachel DePencier, Maya N. Demby, Andrew S. Azman, Elizabeth C. Lee

**Affiliations:** 1 Johns Hopkins University Bloomberg School of Public Health, Baltimore, Maryland, USA; 2 University of Geneva, Geneva, Geneva, Switzerland; 3 Geneva University Hospitals, Geneva, Geneva, Switzerland

**Keywords:** cholera, surveillance, outbreaks

## Abstract

**Background:**

Cholera remains a significant public health challenge in Africa, where outbreaks routinely strain public health and healthcare systems. Cholera surveillance time series data could be used to inform the efficient distribution of resources like oral cholera vaccine (OCV) and emergency response personnel, spur research on the impacts of different cholera control activities, and investigate longer-term epidemiologic patterns in cholera dynamics in this region. However, public reporting of cholera surveillance data historically has been sporadic and often limited to outbreak periods, thus limiting the availability of these useful time series datasets. We sought to fill this gap by preparing, cleaning, and processing weekly consecutive time series and outbreak-specific time series from 2010 to 2023 for African countries from heterogeneous data contributed to a global cholera surveillance database.

**Methods:**

Cholera incidence data in Africa from 2010 to 2023 on suspected cases, confirmed cases, and deaths were compiled from public and non-public cholera surveillance from multiple sources, including ministries of health, World Health Organization, Médecins Sans Frontières, UNICEF, and other sources. Data were processed by aggregating daily records to weekly levels, averaging duplicate entries, filling gaps and surrounding weeks with zero cases, aligning epidemic weeks, adjusting population data, and removing identifying geographic information to preserve confidentiality as appropriate. Outbreaks were subsequently extracted using a systematic definition and summary statistics were produced by spatial scale and population density. Summary outbreak metrics were compared to previously published cholera outbreak datasets for data validation.

**Conclusions:**

The unified surveillance and outbreak datasets provide an extensive compilation of reported cholera activity in Africa from 2010 to 2023, serving as a valuable resource for long-term control planning and early warning systems. Public health researchers can also leverage these datasets to analyze outbreak dynamics, anticipate resource needs, and assess the theoretical impact of control strategies.

## Introduction

Cholera remains a significant global health challenge, disproportionately affecting populations with limited access to water, sanitation, and hygiene (WASH). ^1^ In recent years, cholera outbreaks have continued to appear in both endemic regions and areas that have not recently reported cases, despite heightened cholera control activities. ^2– 5^ Many of these recent outbreaks, particularly in Africa, have been marked by high case fatality risks (CFRs), straining public health and healthcare systems and increasing the demand for critical resources such as oral rehydration solution (ORS) and oral cholera vaccine (OCV). ^6^

Accessibility to continuous cholera surveillance data is critical for improving our understanding of cholera epidemiology and developing data-driven control strategies. Case-based time series can contribute to analyses on long-term patterns in transmission dynamics, endemicity, and seasonality, ^5, 7^ identify early warning signals for timely outbreak detection, and anticipate resource needs for outbreak response. ^8– 10^ These data can also be leveraged in scenario modeling to assess the impact and cost-effectiveness of different control activities like implementing diagnostic testing in surveillance, OCV campaigns, or WASH interventions. ^10– 15^ Such models can also be used to set public health targets or identify benchmarks in target product profiles (TPPs) for diagnostics and vaccines. ^16^ Despite the significance of high-quality surveillance data in guiding these control efforts, cholera surveillance has been neither systematically reported nor widely available. ^17– 23^ Weekly cholera surveillance data for Africa has only begun to be reported by the World Health Organization (WHO) in 2023, and more historical data are critically needed to advance research and policy on cholera control strategies. ^24^

Here, we present four historical datasets of case-based surveillance of suspected cholera at national and subnational levels across Africa from 2010 to 2023: a weekly continuous suspected cases dataset and a subset of the weekly dataset representing only cholera outbreak periods from completely public data sources (i.e., *public surveillance dataset* and *public outbreak dataset*), and two analogous datasets from a mix of public and non-public data sources with masked location information (i.e., *location-masked surveillance dataset* and *location-masked outbreak dataset*). The public datasets are accompanied by a linkable dataset of shapefile geometries. All data products were derived from a cholera incidence database that integrates surveillance data from diverse sources, including situation reports, linelists, and other public records. By alleviating the gaps in non-systematic cholera surveillance and outbreak reporting in Africa, these datasets contribute to the global efforts to mitigate the burden of cholera and strengthen outbreak preparedness and response capabilities.

## Methods

### Collection and preparation of weekly time series data

Cholera incidence data were compiled from a combination of public and non-public sources within the continuously updated Cholera Taxonomy database ( https://cholera-taxonomy.middle-distance.com/). ^25^ To ensure the completeness and validity of the data in this database, systematic data entry and validation activities are conducted regularly. This includes a comprehensive data entry system that integrates surveillance data from diverse sources such as Médecins Sans Frontières and ReliefWeb, along with periodic data sweeps from authoritative public sources such as the UNICEF Cholera Platform, WHO and the WHO Regional Office for Africa to ensure the inclusion of high-quality surveillance data, such as weekly subnational observations. ^24, 26, 27^ Additionally, the database undergoes regular updates to replace outdated records with the latest situation reports.

A standardized system of location hierarchies and shapefile geometries is also maintained in Cholera Taxonomy. Location auditing was performed systematically to verify location names (called “Locations” in the database), a location’s level in the administrative hierarchy, and location boundaries. Locations were linked to shapefiles for the time period that a given boundary was valid (called “Location Periods” in the database), thus enabling documentation of changing boundaries over time. Shapefiles were sourced primarily from centralized and curated data sources, including shapefiles The Humanitarian Data Exchange, Global Administrative Areas (GADM), and geoboundaries. ^28– 30^

**Figure 1.**
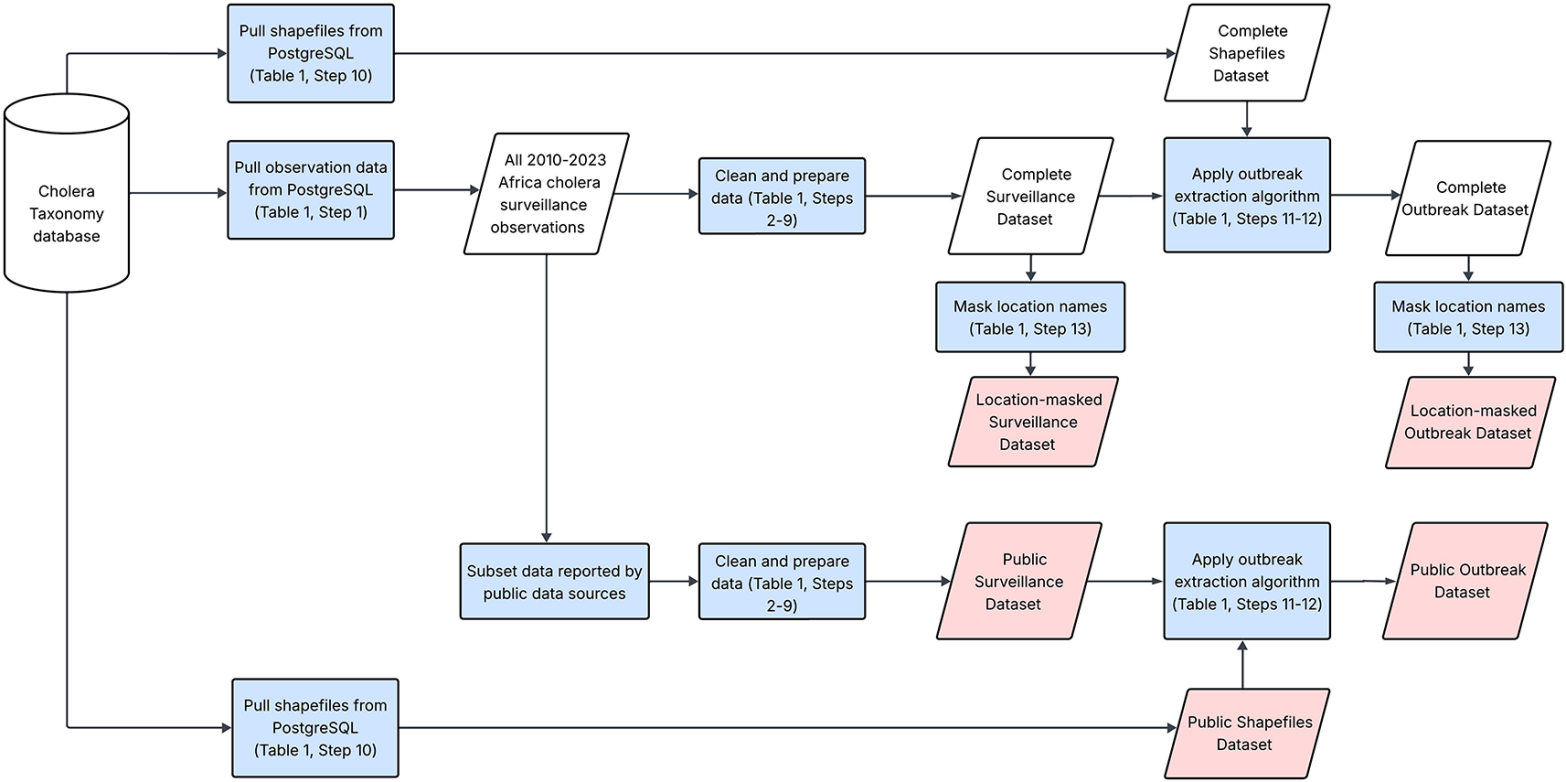
Data processing flowchart for shared outputs. This figure illustrates the general workflow for pulling and cleaning data from the Cholera Taxonomy database. The cylinder represents the Cholera Taxonomy database, which contains global cholera surveillance data. Blue rectangles highlight data processing steps and parallelograms represent datasets. The pink parallelograms show the published data outputs.

Daily and weekly cholera incidence data across Africa from January 1, 2010 to December 31, 2023 on suspected cases, confirmed cases, and deaths were extracted from the Cholera Taxonomy database in November 2023. These data were then processed into weekly consecutive time series— hereafter, *surveillance datasets* —using functions in the OutbreakExtractR package (release v1.0, https://github.com/HopkinsIDD/OutbreakExtractR/tree/main) ( Table 1, Figure 1). In brief, daily cholera data were aggregated to a weekly level and combined with the original weekly cholera data. Weeks with duplicate observations were averaged and observations with different epidemiologic week starting days (Sunday versus Monday start) were shifted to the nearest week that matched the uniform start dates. Weeks with missing data in between the first and last reported observations and the eight weeks before and after the first and last reported observations were assumed to have zero suspected cholera cases (henceforth, “implicit zero observations”). Observation locations are available at country level (ADM0) down to third-level administrative unit scale (ADM3).

**Table 1.** Overview of data preparation and corresponding R functions in *OutbreakExtractR* package. The columns describe the data processing functionality, *OutbreakExtractR* function name, and the specific data cleaning steps performed.

Data processing	R function name	Detailed description
Step 1. Pull cholera data from the Cholera Taxonomy database. If creating the public datasets, filter observations extracted from public data sources	*Not Applicable*	*The database query is performed outside of R.*
Step 2. Clean extracted cholera data	clean_psql_data	Wrapper function that calls multiple cleaning functions: a. Clean location names; b. Standardize descriptive columns; c. Filter out non-primary observations (The primary observations are those stratified by space and time, representing the total number of cases reported in each location during a specific time period); d. Average duplicate observations; e. Add metadata fields, including spatial and temporal scale for each observation.
Step 3. Select relevant observations	observation_filter	Filter out observations based on user-defined settings: a. Study time period; b. Study location; c. Flag to remove observations without suspected or confirmed cholera cases; d. Minimum case threshold; e. Spatial scale (e.g., country, admin1, admin2, etc); f. Temporal scale (e.g., daily, weekly, etc).
Step 4. Convert daily observations to weekly ones	observation_aggregator	If there are any daily data, sum the daily counts of suspected cholera cases, confirmed cholera cases, and deaths to get the total for each week (days with missing data within each week are assumed to have 0 cases or deaths).
Step 5. Average any duplicate observations	average_duplicate_observations	a. For observations with identical locations and overlapping epidemic weeks, calculate the mean of count-based surveillance fields (i.e., suspected cholera cases, confirmed cholera cases, and deaths); b. After averaging, retain only one observation per location and epidemic week by replacing the duplicates with the new averaged entry.
Step 6. Standardize epidemic weeks	set_uniform_wday_start	a. Define the desired uniform day of the week as the start day of each weekly observation; b. Adjust observation dates to begin on the user-specified weekday.
Step 7. Fill missing location periods based on other location period-linked locations in the dataset	fill_missing_lps	a. Identify observations with missing shapefiles (indicated by location_period_id field); b. Search the dataset for other observations from the same location that contain a valid location_period_id; c. Assign the most recent alternative location_period_id for the same location to the observation with a missing shapefile.
Step 8. Remove locations with only one observation	*Not Applicable*	*This step is performed outside an R function.*
Step 9. Fill implicit weekly zeros for suspected cholera cases within the 8 weeks before the earliest date and 8 weeks after the latest date for each location	fill_phantom_zeroes	a. For each location, identify all corresponding observations; b. Before the first and after the last recorded observation for each location, insert 8 weeks of additional time points. For these new time points, fill suspected cases with 0 to ensure continuity in the time series (implicit zeroes); c. Add a new “phantom” column to indicate whether the observation was explicitly reported or an implicit zero (phantom) observation.
Step 10. Pull shapefiles for all the locations with location_period_id from an internal server	get_shp	a. Log into the internal server; b. Pull shapefiles from the database; c. For non-administrative regions composed of multiple sub-regions, individual shapefiles were unified into a single shapefile.
Step 11. Estimate population data for all the locations using the extracted shapefiles	get_pop	a. For each country and year, calculate the adjustment factor by comparing the total country-level population derived from the Worldpop raster to the corresponding population estimate from the UN (i.e., WPP2020 dataset) for that year. The formula would be: Population Adjust Factor = UN Population Estimate/Worldpop Raster Population Sum; b. Multiply the relevant population column by the calculated adjustment factor for all observations from that country and year; c. Use the most recent population adjustment factor for post-2020 observations.
Step 12. Extract outbreaks	identify_outbreaks	a. Apply unified outbreak definition to all observations; b. Calculate the outbreak threshold for all observations; c. Identify the outbreak start; d. Identify the outbreak tail; e. Extract outbreaks.
Step 13. If working with all data (public and non-public), mask geographic information	*Not Applicable*	*This step is performed outside an R function.* Replace location names with random alphabetical letters for datasets containing non-public observations.

### Preparation of population data

Population data were essential for calculating outbreak thresholds. ^10^ The spatial distribution of population estimates were obtained from the WorldPop 100 m by 100 m gridded dataset via the wpgpDownloadR R package, and the gridded estimates were scaled such that country-level estimates matched 2022 United Nations World Population Prospects projections when using geoboundaries shapefiles. ^30– 33^ The spatial distribution of 2021-2023 was assumed to be the same as that in 2020, as 2020 was the latest available WorldPop gridded dataset. Population estimates were calculated as the sum of the area-weighted overlap between grid cells and the location shapefile. Five locations with zero cases reported and an estimated population of zero were removed from the dataset.

### Extraction of outbreak dataset

A systematic and location-specific cholera outbreak definition was applied to the surveillance datasets to create the *outbreak datasets* (Step 12 in Table 1, Figure 1). The outbreak definition that was applied was previously described in Zheng et al. (2022), which analyzed cholera outbreaks in sub-Saharan Africa from 2010 to 2019. ^10^ In brief, an outbreak began when the weekly cholera incidence rate surpassed the location-specific outbreak threshold, followed by at least two consecutive weeks of increasing incidence rates. An outbreak ended when the weekly incidence rate went below the outbreak threshold for two consecutive weeks and was followed by a four-week washout period that remained below the threshold. The outbreak threshold was defined as the location-specific average weekly incidence rate over the complete available dataset.

Outbreaks statistics were stratified by subnational scale (ADM1 to ADM3). These included: outbreak threshold, outbreak size (total cases during the outbreak), outbreak duration, time to peak week, proportion of suspected cholera cases reported during the peak week, weekly incidence during the peak week, early outbreak reproductive number (the average reproductive number over the first week of the outbreak), time to observing 50% of total cases (time _cases50_), outbreak attack rate (total cases per 1,000 population), and CFR. The outbreak peak was defined as the week with the maximum number of cases. We also calculated the population-weighted case-fatality risk for each spatial scale.

### Ethics statement

According to the Institutional Review Board (IRB) at the Johns Hopkins Bloomberg School of Public Health (BSPH), the surveillance and outbreak datasets constructed from the Cholera Taxonomy database were exempt (BSPH IRB No. 27682).

### Data description

We prepared four time series data files for release. Two products are weekly consecutive time series and outbreak-specific time series of suspected cases from public data sources – the *public surveillance dataset* and *public outbreak dataset*, respectively ( Figure 1). These data are accompanied by a linkable dataset of shapefile geometries ( Figure 1). Columns are provided to enable users to retrieve the original source documents from the Cholera Taxonomy database website by entering the observation collection unique ID (OC UID) into the search bar. ^25^

We also provide weekly consecutive time series and outbreak-specific time series datasets as derived from the complete set of Cholera Taxonomy data sources with masked location names – the *location-masked surveillance dataset* and *location-masked outbreak dataset*, respectively ( Figure 1). Locations were masked in these datasets as some data in Cholera Taxonomy were provided under data sharing agreements that prohibited sharing of individual or identifiable aspects of the data.

**Figure 2.**
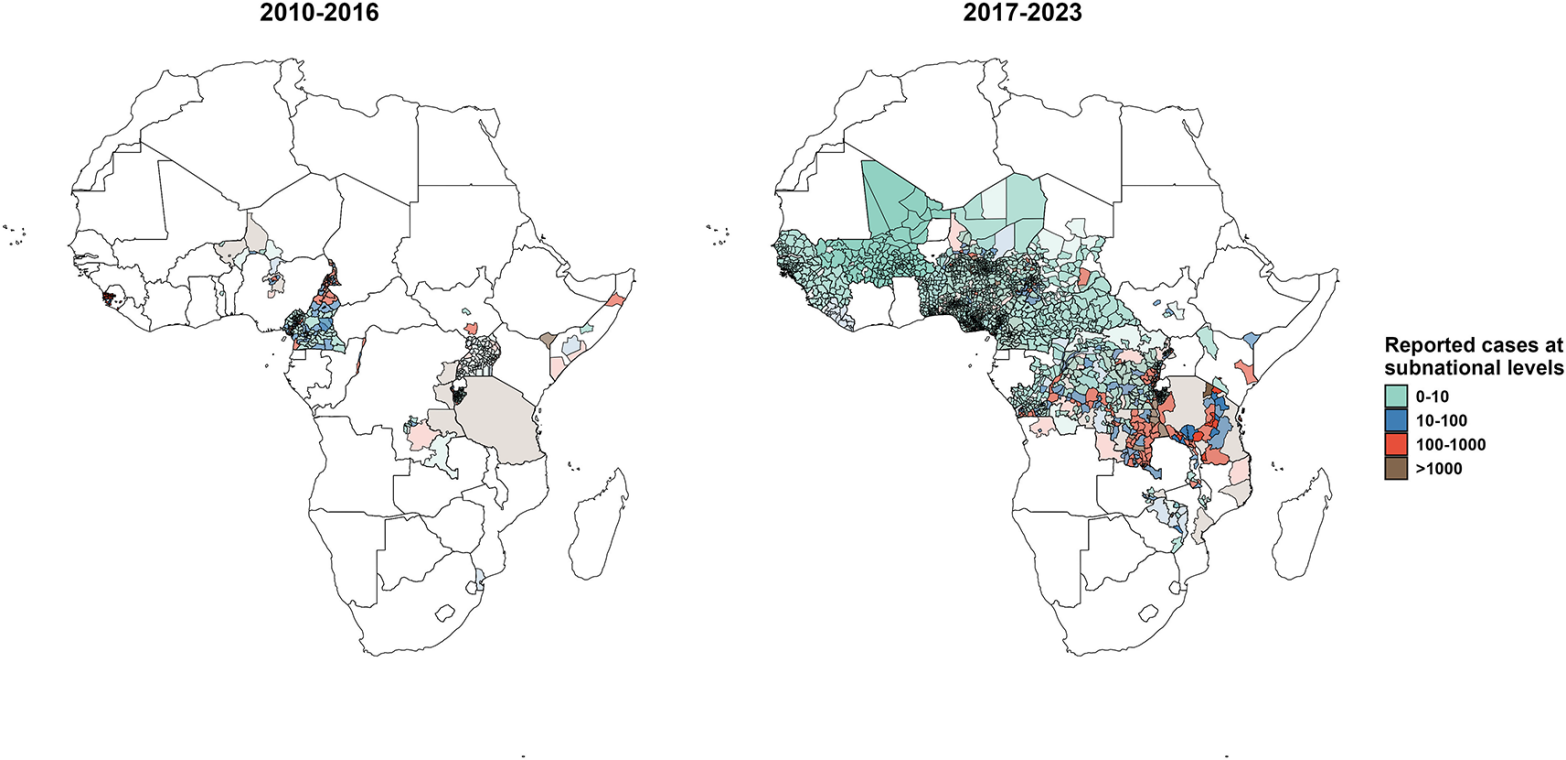
Maps of reported cholera cases at subnational levels from 2010-2016 and 2017-2023. These maps illustrate the spatial distributions of reported suspected cholera cases across subnational administrative units in the public surveillance dataset. Data are represented for 2010-2016 (left) and 2017-2023 (right) and overlapping administrative units are layered such that larger units are in the background and smaller units are in the foreground.

The public surveillance dataset includes 336,380 weekly incidence observations across 732 unique weeks from 2010 to 2023. Of these, 27.4% (92,160 observations) were implicit zero observations, 5.0% (16,909 observations) reported at least one suspected case, and the remaining observations were actively reported zeros. This dataset spans 2,336 unique African regions across multiple spatial scales, including 33 countries, 336 first-level administrative units, 1,781 second-level administrative units, and 187 third-level administrative units ( Figure 2). The location-masked surveillance dataset includes 1,259,495 weekly incidence observations reported in 4,161 unique regions in Africa. Among them, 21.8% (274,903 observations) were implicit zero observations while 5.1% (63,825 observations) reported at least one suspected case.

Based on the public outbreak dataset, a total of 391 cholera outbreaks were identified in 279 unique African regions between 2010 and 2023, including 46 outbreaks at the national level, 109 outbreaks at the first-level administrative units, 218 outbreaks at the second-level administrative units, and 18 outbreaks at the third-level administrative units ( Table 2). According to the location-masked outbreak dataset, 1,668 cholera outbreaks were extracted in Africa from 2010 to 2023. 70 outbreaks were reported at the national level, 205 outbreaks were reported at the first-level administrative units, 1,028 outbreaks at the second-level administrative units, 337 outbreaks at the third-level administrative units, and 28 outbreaks in non-administrative regions ( Table 2).

**Table 2.** Summary of cholera outbreaks reported in sub-Saharan Africa, 2010-2023. This table presents key epidemic metrics for the public and location-masked outbreak datasets by different administrative reporting units.

	Outbreaks reported at first-level administrative units	Outbreaks reported at second-level administrative units	Outbreaks reported at third-level administrative units
**Public Outbreak Dataset**			
Number of outbreaks	109	218	18
Outbreak threshold, weekly incidence per 100,000 people (mean, (median, IQR))	0.7 (0.4, 0.1-0.9)	21.4 (1.2, 0.5-3)	11 (9.1, 5.3-11.5)
Outbreak size, cases (mean, (median, IQR))	2191 (724, 194-2837)	596 (194, 83-496)	269 (173, 84-421)
Epidemic durations, weeks (mean, (median, IQR))	18 (15, 10-23)	18 (16, 11-22)	18 (14, 13-20)
Time to epidemic peak, weeks (mean, (median, IQR))	7 (5, 3-8)	7 (4, 3-7)	5 (3, 3-5)
Proportion of suspected cases reported during the peak week (%) (mean, (median, IQR))	20.7 (17.7, 12.5-28.1)	26.3 (21.1, 15.8-32.6)	23.2 (21.7, 17.3-24.7)
Weekly incidence during the peak week per 1,000 people (mean, (median, IQR))	0.1 (0, 0-0.1)	1.8 (0.3, 0.1-0.8)	1.3 (0.8, 0.4-1)
Early outbreak reproductive number (mean, (median, IQR))	1.8 (1.7, 1.6-2)	1.9 (1.8, 1.7-2)	1. 9 (1.9, 1.7-2)
Time to observing 50% of total cases (mean, (median, IQR))	7.8 (6, 4-9)	7.1 (5, 4-8)	7.1 (5, 4-6.8)
Attack rate per 1,000 people (mean, (median, IQR))	0.6 (0.2, 0.1-0.7)	11.6 (1.2, 0.5-3.5)	6.1 (3.7, 1.8-5.1)
Outbreaks with reports of deaths	83	157	18
CFR (%) (mean, (median, IQR))	2.4 (1.9, 0.3-3.2)	3.1 (1.9, 0.4-4.4)	0 (0, 0-0)
Population-weighted CFR (%)	2.6	2.6	0
**Location-masked Outbreak Dataset**			
Number of outbreaks	205	1028	337
Outbreak threshold, weekly incidence per 100,000 people (mean, (median, IQR))	1 (0.4, 0.1-1)	38.7 (0.9, 0.3-2.8)	13.8 (0.8, 0.3-7.3)
Outbreak size, cases (mean, (median, IQR))	2114 (720, 242-2362)	496 (208, 85-486)	251 (107, 42-286)
Epidemic durations, weeks (mean, (median, IQR))	19 (15, 10-23)	17 (15, 10-22)	14 (12, 9-17)
Time to epidemic peak, weeks (mean, (median, IQR))	8 (5, 3-8)	6 (4, 3-7)	5 (3, 3-5)
Proportion of suspected cases reported during the peak week (%) (mean, (median, IQR))	20.8 (17.7, 13.7-26.1)	25.6 (22.2, 15.5-32.7)	29.5 (27.8, 19.5-37.5)
Weekly incidence during the peak week per 1,000 people (mean, (median, IQR))	0.2 (0.1, 0-0.2)	4.3 (0.2, 0.1-0.5)	2.2 (0.3, 0.1-1.1)
Early outbreak reproductive number (mean, (median, IQR))	1.8 (1.7, 1.6-2)	1.9 (1.8, 1.6-2.1)	1.9 (1.9, 1.7-2.1)
Time to observing 50% of total cases (mean, (median, IQR))	7.9 (6, 4-9)	6.6 (5, 3-8)	5.5 (4, 3-6)
Attack rate per 1,000 people (mean, (median, IQR))	1 (0.3, 0.1-1.1)	20 (1, 0.3-2.8)	9.7 (1.2, 0.4-4.8)
Outbreaks with reports of deaths	180	990	318
CFR (%) (mean, (median, IQR))	1.9 (1.2, 0.3-2.7)	2.8 (1.7, 0.3-3.9)	1.1 (0, 0-1.3)
Population-weighted CFR (%)	1.8	2	1.3

### Limitations

While these datasets constitute one of the most extensive collections of cholera surveillance data in Africa from 2010 to 2023, there are several challenges. For one, we note that these datasets are based on reported suspected cholera cases and include data from multiple case definitions. Although suspected cases serve as the primary reported metric in cholera surveillance, the substantial variability in positivity rates among suspected cases and inconsistencies in case definitions may limit the ability of the datasets to accurately capture the true cholera burden. ^34^ Given the disparate sources of data and reporting at multiple spatial scales, the dataset includes discrepancies and potential duplicate reporting when observations overlap in space and time. Further, gaps in surveillance and reporting and limited availability of weekly data mean that the dataset may not be completely representative of all cholera reports in Africa during this period. To alleviate these gaps, we assigned zero cases to weeks without reported data. However, comparison with outbreaks characterized in our previous outbreak paper, where “implicit zeroes” were not applied, suggested that this “implicit zero cases” assumption may lead to lower outbreak thresholds, larger outbreak sizes, and extended outbreak periods in some regions ( Table 4). ^10^ Additionally, outbreaks occurring at the beginning or end of the analysis period may have been truncated when we applied the outbreak extraction algorithm to the 2010-2023 period. Despite systematic auditing of location names and shapefiles, the large geographic and temporal scales of the database pose challenges to ensuring the geographic accuracy in our datasets. Population estimates and therefore outbreak thresholds and attack rate metrics depend on the quality of the gridded WorldPop source we used, and are further constrained by the availability and validity of shapefiles. Population estimates after 2020 were derived by applying the 2020 WorldPop spatial distribution to country-level population totals for post-2020 years. While UN population data were used to scale national totals for all years, the absence of single-year WorldPop rasters beyond 2020 led to the assumption that the spatial distribution of post-2020 populations remained stable relative to 2020, which may not match reality.

### Dataset validation

External validity

We compared descriptive statistics from the location-masked surveillance and outbreak datasets to those reported in an independent study, Koua et al. (2025) on cholera outbreaks in the WHO African region (WHO AFRO) from 2000 to 2023. ^35^ These statistics represented the number of WHO AFRO countries reporting cholera outbreaks per year and the annual case fatality risk ( Table 3). For the purpose of the table, we grouped outbreaks according to the year of outbreak start. As cholera outbreak definitions in Koua et al. (2025) were not systematically defined, we compared their counts to two metrics from our datasets – number of countries reporting at least one suspected cholera case in the location-masked surveillance dataset, and number of countries reporting outbreaks in the location-masked outbreak dataset. CFR definitions also differed between the two sources. Koua et al. (2025) appeared to report the total ratio of reported deaths and cases in a given year and we calculated the population-weighted average CFR across all outbreaks that actively reported death counts (including zero deaths) from our location-masked outbreak dataset.

**Table 3.** Comparison to 2010-2023 outbreak metrics in sub-Saharan Africa in Koua et al. (2025). This table compares the number of countries affected by cholera outbreaks annually and the annual CFRs between Koua et al. (2025) and the location-masked datasets. The number of countries with cholera death reporting, noted in parentheses in the fourth column, represents the subset of data used to calculate the annual population-weighted CFR in the location-masked outbreak dataset (sixth column).

Year	Number of countries with outbreaks in Koua et al. (2025)	Number of WHO AFRO countries with at least one suspected case in location-masked surveillance dataset	Number of WHO AFRO countries with outbreaks in location-masked outbreak dataset (countries with reports of deaths)	Annual CFR (%) in Koua et al. (2025)	Annual population-weighted CFR (%) in WHO AFRO countries in location-masked outbreak dataset
2010	21	16	11 (9)	3.0	3.6
2011	25	17	11 (9)	2.8	3.3
2012	26	19	14 (13)	1.9	2.0
2013	21	16	8 (7)	2.5	1.3
2014	18	15	11 (8)	1.8	3.1
2015	15	15	11 (10)	1.3	5.9
2016	16	20	11 (10)	2.2	1.9
2017	13	20	13 (11)	2.1	0.8
2018	16	22	12 (10)	2.1	1.4
2019	14	19	10 (9)	1.7	1.0
2020	12	10	6 (5)	1.7	1.3
2021	20	11	5 (5)	3.0	3.8
2022	17	15	7 (6)	2.2	1.9
2023	17	5	1 (0)	1.5	-

When comparing the number of countries with at least one suspected case in the location-masked surveillance dataset and the number reporting outbreaks in Koua et al. (2025), there was little concordance between the two sources and no source reported systematically more countries with cholera ( Table 3). ^35^ However, Koua et al. (2025) did systematically have more countries reporting outbreaks than our location-masked outbreak dataset. There were also differences in the reported annual CFRs between the two studies ( Table 3), but no source systematically reported higher CFRs.

Discrepancies in these metrics are due to different data sources, missingness, and outbreak extraction criteria. Most locations in our location-masked surveillance dataset were missing years of data between 2010 and 2023. Substantial data in the Cholera Taxonomy database could not be compiled to a weekly scale and were thus excluded from these data products. Limited data from the Integrated Disease Surveillance and Response (IDSR) system were present in the Cholera Taxonomy database (only 5 countries in the WHO AFRO region between 2006 and 2022), whereas this was the primary source of the Koua et al. (2025) cholera data. ^35^ Further, there was limited weekly reporting of cholera deaths in Cholera Taxonomy, which meant that CFR could only be calculated for a subset of outbreaks. Another source of discrepancy is different methods for defining cholera outbreaks. Our dataset applied a standardized outbreak definition based on historical weekly incidence, while Koua et al. (2025) defined cholera outbreaks according to what was reported by the WHO event management system. ^35^ Both approaches may be subject to missingness with different biases, and relative to the other dataset, our approach may particularly exclude outbreaks that are particularly small, short, or in cholera-endemic regions. We are not certain what biases may exist with regards to outbreaks recorded by the WHO event management system.

Internal validity

Data from 2010-2019 in the location-masked outbreak dataset was validated to our team’s previously published 2010-2019 African cholera outbreak dataset by comparing outbreak locations, start and end dates, and outbreak metrics. ^10^ Outbreaks from the two datasets were classified into three types of outbreak pairs: 1) outbreaks with identical start and end dates ( *identical outbreaks*), 2) outbreaks that started within 4 weeks of the original outbreak start date ( *outbreaks with similar start*), and 3) other outbreaks in the same location with overlapping time periods ( *overlapping outbreaks*).

We identified 702 outbreaks with identical start and end dates, 206 outbreaks with similar start dates, and 13 overlapping outbreaks. The outbreak metrics for outbreaks reported at the second-level administrative units in different categories are summarized in Table 4, showing similar distributions across all metrics and categories, and confirming the consistency and validity of this dataset. We also noted 78 outbreaks from the original dataset not found in our new dataset, and 485 outbreaks identified in the new dataset which were not reported in the original publication. ^10^

**Table 4.** Comparison to 2010-2019 outbreak metrics in Zheng et al. (2022). This table compares key outbreak metrics from Zheng et al. (2022) and the location-masked outbreak dataset. Where appropriate, we reported the mean, median, and interquartile range (IQR) across all outbreaks in a given category.

Metrics	Identical outbreaks from Zheng et al. (2022)	Identical outbreaks in the location-masked outbreak dataset	Outbreaks with similar start from Zheng et al. (2022)	Outbreaks with similar start from the location-masked outbreak dataset	Overlapping outbreaks from Zheng et al. (2022)	Overlapping outbreaks from the location-masked outbreak dataset
Number of outbreaks	485	485	142	142	11	11
Outbreak threshold, weekly incidence per 100,000 people (mean, (median, IQR))	14.2 (0.6, 0.2-2)	2.8 (0.5, 0.2-1.9)	16.6 (2.1, 0.4-5.3)	4 (1.1, 0.3-3.6)	2.3 (0.3, 0.1-2.6)	2.4 (0.3, 0.2-3)
Outbreak size, cases (mean, (median, IQR))	445 (182, 71-422)	445 (182, 71-422)	563 (224, 77-492)	624 (254, 90-627)	172 (76, 44-106)	281 (151, 56-314)
Epidemic durations, weeks (mean, (median, IQR))	16 (13, 9-19)	16 (13, 9-19)	14 (12, 8-17)	17 (14, 10-21)	25 (19, 12-34)	27 (25, 18-28)

Discrepancies between the two datasets may be explained by several factors. Since the original publication, we identified and processed new surveillance data for the 2010-2019 period, refined the data preparation procedure to include outbreaks spanning multiple administrative regions, and harmonized observations into standardized epidemiologic weeks across data sources. We also scaled population data to match annual, country-level UN population estimates, which led to modifications in outbreak thresholds, thus shifting potential outbreak timing. Further, we added a new data preparation step that added “implicit zero cases” to weeks without reported data. This change systematically lowered the outbreak threshold in many locations and enabled more locations to meet outbreak start and end criteria by mitigating the effect of data truncation on outbreak extraction. We anticipated that this change would generally increase the number of outbreaks identified and extend the duration of some outbreaks, which is what we observed ( Table 4).

## Ethics statement

According to the Institutional Review Board (IRB) at the Johns Hopkins Bloomberg School of Public Health (BSPH), the surveillance and outbreak datasets constructed from the Cholera Taxonomy database were exempt (BSPH IRB No. 27682).

## Software availability

Source code for OutbreakExtractR R package is available from: https://github.com/HopkinsIDD/OutbreakExtractR/tree/main. Archived software for OutbreakExtractR v1.0 is available from: https://doi.org/10.5281/zenodo.15319642. License: GPL-3.0.

## Data Availability

Underlying data is available at OSF under a CC-BY Attribution 4.0 International license: Lee EC, Zheng Q. Cholera surveillance time series in Africa from 2010 to 2023 [Internet]. OSF; 2025. Available from: osf.io/2ncf7 ^36^ This project contains the following datasets which may be opened using R:
•
**
Public_surveillance_dataset.rds**: cholera surveillance time series from public data sources. •
**
Public_outbreak_dataset.rds**: cholera outbreak time series from public data sources. •
**
Location_masked_surveillance_dataset.rds**: location-masked cholera surveillance time series. •
**
Location_masked_outbreak_dataset.rds**: location-masked cholera outbreak time series. •
**
Public_surveillance_shapefiles.rds**: shapefiles associated with all locations in the public surveillance and public outbreak datasets. •
**data_dictionaries.xlsx**: includes a README file and describes the columns of each dataset. Data are available under the terms of the Creative Commons Attribution 4.0 International license (CC-BY 4.0).
